# Factors influencing the uptake of neonatal bereavement support services – Findings from two tertiary neonatal centres in the UK

**DOI:** 10.1186/s12904-016-0126-3

**Published:** 2016-06-29

**Authors:** Jayanta Banerjee, Charanjit Kaur, Sridhar Ramaiah, Rahul Roy, Narendra Aladangady

**Affiliations:** Neonatal Unit, Homerton University Hospital NHS Foundation Trust, Homerton Row, London, E9 6SR United Kingdom; Centre for Paediatrics, Barts and the London School of Medicine and Dentistry, Queen Mary University of London, London, UK; Neonatal Unit, Imperial College Healthcare NHS Trust, London, UK; Neonatal Unit, Norfolk and Norwich University Hospital NHS Trust, Norwich, UK; Department of Paediatrics, SDM Medical College and Hospitals, Dharwad, India

**Keywords:** Neonatal, Bereavement follow up, Death, Ethnicity, Socio-economic, Autopsy

## Abstract

**Background:**

Research on perinatal bereavement services is limited. The aim of the study was to compare the uptake of bereavement support services between two tertiary neonatal units (NNU), and to investigate influencing factors.

**Method:**

The medical and bereavement records of all neonatal deaths were studied from January 2006 to December 2011. Data collected included parent and baby characteristics, mode of death, consent for autopsy and bereavement follow-up. The categorical data were compared by chi-square or Fisher’s exact test and continuous data by Wilcoxon signed-rank test; a multivariable regression analysis was performed using STATA 12.0.

**Results:**

The neonatal deaths of 297 babies (182 in NNU1 and 115 in NNU2) with full datasets were analysed. Baby characteristics were similar between units except for lower median gestational age in NNU1 (*p* = 0.03). Significantly more NNU1 parents were non-Caucasian (*p* < 0.01), from lower socio-economic status (*p* = 0.01) and had previous stillbirth/miscarriage (*p* = 0.03). More babies had care withdrawn in NNU2 (*p* < 0.01). A significantly higher proportion of parents from NNU1 (61 %) attended bereavement follow-up compared to NNU2 (34 %; *p* < 0.01).

On multivariable analysis, significantly more parents who were married or co-habiting (*p* = 0.02) and consented for an autopsy (*p* = 0.01) attended bereavement services.

**Conclusion:**

Uptake of bereavement services varied between the two NNUs, which could be due to differences in the ethnic and socio-economic mix of the population. Significantly more parents who were married or co-habiting, or consented for autopsy, attended bereavement follow up services.

## Background

With considerable advances in obstetric and neonatal care over the past two decades, parents now anticipate successful outcomes of high risk pregnancies and extreme premature births, expecting their children to survive to adulthood; the loss of an expected child causes severe distress. There were 3558 stillbirths and 1990 neonatal deaths in England in 2012 [1]. Recent data from the Maternal, Newborn and Baby Clinical Outcome Review Programme, MBRACE-UK has shown that the stillbirth rate is currently 4.2 per 1000 pregnancies and neonatal death rate is 1.8 per 1000 deliveries [2]. Apart from the emotional trauma of physical separation from their baby, perinatal loss also encompasses social loss of their role as parents. This can have a significant impact on their physical and psychological wellbeing [3]. It has been well documented that parental bereavement is particularly intense, complicated and long lasting, with major symptom fluctuations over time [4]. Health professionals dealing with parents need to identify if there are factors in the management of the loss that facilitate or hinder recovery, and whether there are specific interventions that can be offered that will help parents and families.

Bereaved parents have expressed the need for comprehensive information about their child's illness and death, the opportunity to provide feedback about their hospital experience, and emotional support, mainly in the form of reassurance and the sense of being cared for during their crisis [5–9]. Traditionally the comfort and support of bereaved parents are provided by family and friends, religious representatives, various charities such as SANDS (Stillbirth and Neonatal Death charity) and hospital. In most hospitals in the UK bereavement support is provided by a multi-professional team. It is free and includes bereavement counselling and follow up appointments; this is provided by either a neonatologist or an obstetrician, or both, and facilitated by a bereavement midwife or nurse. Care providers such as children’s hospices provide perinatal palliative care to babies and their families in situations where newborns require redirection of care. The hospices are charity funded in the UK, and may also provide one to one counselling and bereavement support to the parents [10]. A report by SANDS in 2010 has shown that designated bereavement support midwives were present in only 47 % of the hospitals in the UK. The number of bereavement support appointments varied from one to several; these were mainly co-ordinated by bereavement support midwives [11]. The cause of the baby's death (if known), circumstances leading to it and results of autopsy and other pending investigations are discussed and explained to parents. Any implications for future pregnancies are also discussed [12]. This relieves parents of feelings of guilt and helps them cope with their grief by bringing closure [13]. However, not infrequently, it has been observed that parents either refuse, or do not attend, counselling or follow up appointments.

We have shown previously that the parents’ ethnic and cultural background influences their decision to withdraw life sustaining treatment (LST) [14]. However, the relationship between the baby, parental characteristics, mode and cause of death, autopsy and uptake of bereavement services in the neonatal practice in the UK has not been studied. The objectives of the present study were to compare the uptake of bereavement support services by parents at two tertiary level neonatal units catering to ethnically different populations in the UK and to investigate whether any baby or parental characteristics influence the uptake of these services within and between the two centres.

## Methods

The study was conducted at Homerton University Hospital and Norfolk and Norwich University Hospital over a six year period. Both the hospitals are tertiary neonatal referral centres, in addition Norfolk and Norwich University Hospital has onsite regional Paediatric surgical services. Homerton caters to an ethnically and culturally diverse inner city population, while Norfolk and Norwich have a predominantly Caucasian population. Both have a robust bereavement support program with dedicated perinatal bereavement support midwives established for over 10 years. The bereavement support midwife co-ordinates the care following a stillbirth or the death of a baby. They meet with the family soon after the death and provide initial bereavement support, including organising the death certificate, funeral arrangements and spiritual support. Psychology input is not readily available at either neonatal unit studied, but appropriate referral is made by the bereavement midwives where this is required. The maternal General Practitioner (GP; family doctor) is informed regarding the death of the baby and the wellbeing of the parents, particularly if they need additional help. An appointment letter is sent to the parents to attend the bereavement support clinic approximately 6 weeks after the baby's death. Parents also have the option to contact the bereavement midwives (over the phone or in clinic) while waiting for the bereavement follow up appointment. The bereavement follow up clinic is usually led by a Consultant Neonatologist along with a bereavement midwife; occasionally this is conducted jointly with an obstetrician as well. The main purpose of the bereavement follow up clinic is to clarify for parents the clinical management and progress of the baby, cause of death and autopsy findings where applicable, and to answer parents’ questions. Parents’ wellbeing and need for additional help (e.g. emotional or psychological) are also discussed.

Information on all neonatal deaths in the neonatal unit and labour ward at both centres from January 2006 to December 2011 were collected. The babies whose notes were not traceable or had an incomplete dataset were excluded from the study. Details of babies studied were collected from medical records and the Standardised Electronic Neonatal Database (SEND). Baby characteristics such as inborn (born in the same hospital) or outborn (transferred after birth from a different hospital), gestational age (GA), birth weight (BWt), sex, singleton/twins, natural or assisted conception, and number of siblings were collected. Specific data relating to the death of the baby included place of death, postnatal age at the time of death, mode of death, whether natural or limiting Life Sustaining Treatment (LST), and parents consent for autopsy. Parental characteristics such as maternal age, standard social classification based on the 2010 coding index [15], relationship status, ethnicity and previous neonatal deaths or stillbirths were collected. Details of bereavement follow up for each neonatal death were accessed from medical records, as well as records of the bereavement midwives, to investigate attendance.

Data were analysed to compare the uptake of bereavement support services between the two participating centres and to determine the relationship between parental and baby characteristics affecting uptake of bereavement support services. Discrete variables are reported as n (%) and were compared using Chi square or Fishers Exact test. Continuous variables are reported as mean (standard deviation) or median (inter-quartile range) and were assessed using Students t test or Wilcoxon rank test according to distribution. Multivariable analysis using binary logistic regression analysis was performed including variables that were significant at 10 % (*p*-value <0.1) after a univariate analysis. The dependent variable was uptake of bereavement support services (yes vs. no). For the multivariable analysis, apart from the included variables, the following were collapsed into two categories; marriage/co-habiting (yes vs. no), socioeconomic status (skilled vs. non-skilled) and ethnicity (Caucasian vs. others). A p-value of 0.05 was considered significant. STATA 12.0 (STATA Corp LP, US) was used for all analysis.

## Results

During the six year study period 308 babies died in the two units [191 in Neonatal unit 1 (NNU1) and 117 in Neonatal unit 2 (NNU2)] out of which full datasets of 297 babies (182 in NNU1 and 115 in NNU2) were available for analysis. The rest were excluded due to unavailability of records or missing data. Out of 297 babies with full datasets, 188 (63 %) babies were born at <28 weeks of gestation, 62 (21 %) between ≥28 and <37 weeks and 47 (16 %) were born at ≥37 weeks of gestation. Fifty three percent (*n* = 158) babies were male and 67 % (*n* = 199) babies had birth weight ≤1000 grams. All babies studied died in the hospital (280 in neonatal unit and 17 in the labour ward).

### Uptake of bereavement support services

Fifty percent (*n* = 149) of parents attended bereavement support services. The median time for follow up was 8 weeks (range 4 - 16 weeks) and on all occasions they were seen by a neonatologist and a specialist bereavement support midwife. Twenty five out of the 149 parents were also seen by an obstetrician along with a neonatologist. The parents were seen up to a maximum of 3 times in one case, twice in 24 cases and at least once in 124 cases.

### Comparison between the two centres

The median gestational age was significantly lower in NNU1 (*p* = 0.03). There were no statistically significant differences between the median birth weight (BWt), age of death, number of inborn babies or sex distribution between the two units (Table 1). The proportion of deaths in the neonatal unit compared to the labour ward was similar between the units. A significantly higher proportion of babies died as a result of limiting LST in NNU2 (*p* < 0.01; Table 1). A significantly higher number of parents were Caucasian and from higher socioeconomic class in NNU2 (*p* < 0.01). Significantly more parents experienced previous miscarriage or stillbirth in NNU1 (*p* = 0.03). There was no difference in other parental characteristics studied (Table 2). A significantly higher proportion of parents (61 % vs. 34 %, p <0.01) attended bereavement counselling in NNU 1 (Table 2).

**Table 1:** Comparison of infant characteristics between the two Neonatal Units (NNUs)

	NNU1 (*n* = 182)	NNU2 (*n* = 115)	p value
Gestational age (weeks) ^+^	25 (24 – 30)	27 (24 – 32)	0.03*
Birth weight (grams) ^+^	755 (608 – 1200)	820 (630 – 1670)	0.14*
Inborn ^†^	137 (75 %)	79 (69 %)	0.22^^^
Male/female	91/91	67/48	0.18^^^
Age of death (days) ^+^	4 (1 – 17)	2 (2 - 21)	0.11*
Place of death (NNU/LW)	171 (94 %)/11 (6 %)	109 (95 %)/6 (5 %)	0.32^^^
Mode of death			
Natural ^†^	95 (52 %)	24 (21 %)	<0.01^^^
Withdrawal or withholding of LST ^†^	87 (48 %)	91 (79 %)	
Post mortem examination			
Not done ^†^	148 (81 %)	94 (82 %)	0.99^
Done ^†^	34 (19 %)	21 (18 %)	

*Wilcoxon signed rank test, ^¥^Fisher’s exact test, ^^^Chi square test, ^+^ [median (IQR)]

^†^ [number (%)]

**Table 2:** Comparison of parental characteristics between the two Neonatal Units (NNUs)

	NNU1 (n = 182)	NNU2 (n = 115)	*p* value
Parental relationship ^†^			
Married/cohabiting	135 (74 %)	83 (72 %)	0.71^^^
Single	26 (14 %)	15 (13 %)	
Unrecorded	21 (12 %)	17 (15 %)	
Ethnicity ^†^			
Asian	29 (16 %)	3 (3 %)	<0.01^^^
African and Afro-Caribbean	54 (30 %)	4 (3 %)	
Caucasian	79 (43 %)	92 (80 %)	
Other	16 (9 %)	5 (4 %)	
Not recorded	4 (2 %)	11 (10 %)	
Socio economic distribution ^†^			
Managers and Professionals	35 (19 %)	21 (18 %)	<0.01^^^
Skilled workers	41 (23 %)	55 (48 %)	
Unskilled workers	12 (7 %)	8 (7 %)	
Not working	26 (14 %)	10 (9 %)	
Unrecorded	68 (37 %)	21 (18 %)	
Obstetric history			
Conception (natural /assisted)	171/11	104/11	0.32^^^
No. of previous miscarriages/stillbirths ^+^	0.6 (1.03)	0.3 (0.77)	0.03*
No. of previous neonatal deaths ^+^	0.1 (0.1)	0.2 (0.2)	0.05^¥^
Parents attended bereavement counselling ^†^			
Yes	111 (61 %)	38 (34 %)	<0.01^^^
No	71 (39 %)	76 (66 %)	

*Wilcoxon signed rank test, ^¥^Fisher’s exact test, ^^^Chi square test

^+^[mean (SD)], ^†^ [number (%)]

### Factors associated with parents’ uptake of bereavement support service

On univariate analysis, gestational age and birth weight were significantly associated with parents attending bereavement support services (Table 3). There was no significant association with other baby characteristics, such as place of birth (inborn or outborn OR 1.15; CI 0.72 to 1.83; *p* = 0.56), age at death (OR 1.00; CI 0.99 to 1.00; *p* = 0.88), mode of death (OR 0.96; CI 0.23 to 3.90; p =0.95) or number of siblings (OR 1.04; CI 0.89 to 1.22; *p* = 0.62), and parents attending bereavement appointment. On multivariable analysis none of the baby factors were noted to be significant (Table 3).

**Table 3:** Infant and maternal factors and uptake of bereavement support services (n = 297)

Infant and maternal factors	Univariate OR (95 % CI) P value	Multivariate OR (95 % CI) P value
Gestational age	1.06 (1.01 to 1.11) 0.01	1.03 (0.89 to 1.19) 0.67
Birthweight	1.00 (1.00 to 1.01) 0.01	1.00 (0.99 to 1.00) 0.98
Maternal age	1.03 (0.99 to 1.07) 0.16	0.98 (0.94 to 1.03) 0.48
Married/Co-habiting	0.51 (0.30 to 0.89) 0.01	0.49 (0.27 to 0.88) 0.02
Consent for Autopsy	2.74 (1.44 to 5.21) 0.002	2.92 (1.28 to 6.64) 0.01
History of previous miscarriage	1.44 (0.86 to 2.41) 0.17	1.79 (0.98 to 3.25) 0.06

Maternal age (OR 1.03; CI 0.99 to 1.07; *p* = 0.16), religion (OR 1.05; CI 0.87 to 1.28; *p* = 0.60), ethnicity (OR 1.24; CI 0.89 to 1.76; *p* = 0.20), type of conception (OR 0.78; CI 0.32 to1.86; *p* = 0.57), type of delivery (OR 0.77; CI 0.44 to 1.35; *p* = 0.37), previous neonatal death (OR 0.64; CI 0.11 to 3.91; *p* = 0.63) or social class (OR 0.7; CI 0.44 to 1.1, *p* = 0.12) were not significantly associated with parents’ uptake of bereavement support services. A significantly higher number of parents who were married or cohabiting (*p* = 0.01) and those who consented for autopsy of their babies (*p* = 0.002) attended bereavement support services (Table 3). On multivariable analysis, parents’ relationship (married or co-habiting) and consent for autopsy were significantly associated with parents attending bereavement services. Mothers who had a previous history of miscarriage showed a higher trend for attending bereavement support services but this was not significant (OR 1.79; 95 % CI 0.98 to 3.25; *p* = 0.06; Table 3).

## Discussion

The study has shown that the uptake of bereavement support services by parents differs between neonatal units, and parental characteristics such as relationship status and consent for autopsy influence the uptake of these services. Significantly higher number of parents from NNU1 attended bereavement follow up appointments. A large number of NNU1 parents were of African and Afro-Caribbean ethnic background with a history of previous miscarriage or stillbirth, which might have influenced their bereavement follow-up attendance. The rest of the parental characteristics studied were similar between the two units. Overall between the two units, 50 % of parents attended bereavement follow-up appointments at least once in our study. McHaffie et al reported that 92 % of parents attended bereavement appointments following the death of their babies as result of withdrawal of LST [16]. There was no difference between parents attending bereavement follow-up after the death of a child following withdrawal of LST, or after continuing full intensive care support in our study. Meert et al studied children who died in Paediatric Intensive Care Unit (PICU) and reported that 59 % of parents attended bereavement follow-up appointments [5] similar to the present study. In the current study parents were seen by a consultant neonatologist and a specialist bereavement midwife at all bereavement follow-up appointments. A recent national survey of neonatologists in the UK reported that a consultant neonatologist was involved in providing bereavement support to parents in 80 % of cases and a bereavement specialist in around 50 % [17].

Baby factors such as gestational age, birth weight, sex, number of siblings, place of birth and place and mode of death were not associated with uptake of bereavement follow up appointments. Parents who are married or cohabiting and consented for autopsy were more likely to attend bereavement follow-up appointments in this study. Autopsy remains the gold standard for investigating a perinatal death, and offering and consenting for autopsy of a neonate remains a challenging area in the field of neonatology. Over the years there has been a decline in the number of autopsies in neonatology owing to various factors, such as lack of offering autopsy to parents by neonatologists (60 %), lack of availability of perinatal pathologist (35 %) and a perception that autopsies seldom provide useful new information (37 %) [18]. McHaffie et al have reported that the biggest reason for parental refusal of autopsy is the perception of their baby being mutilated and a feeling that there were no unanswered questions [19]. Those parents who were offered and consented for autopsy in the current study attended the bereavement clinic appointments to further understand the cause of death and for explanation of their unanswered questions. In the current study the uptake of bereavement support appointments was higher for parents in a relationship (married or co-habiting). We speculate that this could be due to effective mutual support between the partners. Further studies are required to identify why some single parents do not attend bereavement follow-ups.

There was no association between parents who attended bereavement appointments and mode of conception, gestational age, birth weight, or postnatal age at death, in this study. No similar published report is available to compare our findings. However, Benfield et al reported that there was no association between grief responses of parents after neonatal death and to birth weight, postnatal age of the baby at death, previous neonatal loss or parental age [20]. Similarly other researchers have found no association for maternal psychological response after perinatal loss, to difficulty in conceiving [21, 22], maternal age [20, 23, 24], sex of the baby [25, 26] and socio-economic status [22, 23, 27]. The majority of these studies were conducted in the US, the UK, Western Europe and Australia and had an under representation or exclusion of ethnic minorities, who might have entirely different cultural or religious practices and beliefs, different ways of coping and varying support systems.

Details about antenatal and neonatal professional communication with parents were not collected in this study, which might have influenced parents’ attendance at bereavement follow-up. In a recent report about the care experienced by parents (n = 248 women) of babies who died before birth or as a newborn across England, around 30 % mothers felt that they were inadequately supported by perinatal staff [1]. Eleven babies (3.5 %) were excluded in the current study because of missing records or data (9 from NNU1 and 2 from NNU2) but this is unlikely to influence the study findings. Another limitation of this study was the lack of data on religious affiliations of parents. However, no association with maternal psychological response after perinatal loss and religion was noticed in previously reported studies [27, 28].

## Conclusion

The uptake of bereavement support services varied between neonatal units, and parents who were married or co-habiting and those who consented for autopsy were more likely to attend the bereavement follow up appointments. Health professionals should implement various techniques to improve the uptake of bereavement support services. Perhaps offering bereavement follow up in a non-hospital based environment would encourage some parents to attend who otherwise want to avoid painful memories. Increasing awareness amongst parents of the presence and utility of such a service may allow them to engage with these support modalities. Involvement of representatives from ethnic minorities and spiritual advisors could help to improve the bereavement support services. Support and care can be individualised based on parents’ social support, ways of coping and any pre-existing stress factors (targeted approach), allowing cognitive and behavioural therapy to be offered to those at risk. Adequate training should be offered to all staff dealing with these parents so that they can offer the right and consistent support to parents without feeling overtly distressed or overwhelmed. Further prospective qualitative and quantitative research is needed in this sensitive and highly complex field to better understand the parents’ perspective with an aim to improve bereavement support services.

## Abbreviations

BWt, birth weight; GA, gestational age; LST, life sustaining treatment; NNU, neonatal unit; NREC, National Research Ethics Committee; RCPCH, Royal College of Paediatrics and Child Health; SANDS, Stillbirth And Neonatal Death charity; SEND, Standardised Electronic Neonatal Database; UK, United Kingdom
